# The Marsden Morbidity Index: the derivation and validation of a simple risk index scoring system using cardiopulmonary exercise testing variables to predict morbidity in high-risk patients having major cancer surgery

**DOI:** 10.1186/s13741-022-00279-8

**Published:** 2022-09-22

**Authors:** Z. Nawoor-Quinn, A. Oliver, R. Raobaikady, K. Mohammad, S. Cone, R. Kasivisvanathan

**Affiliations:** 1 Department of Anaesthesia and Critical Care, The Royal Marsden, London, UK; 2 Department of Surgery and Cancer, St Mary’s Hospital, Imperial College London, London, UK; 3 Department of Anaesthesia, University College London Hospitals, London, UK; 4 The Royal Marsden Hospital and The Royal Marsden NHS Foundation Trust, Fulham Road, Chelsea, London, SW3 6JJ UK

**Keywords:** Cardiopulmonary exercise testing, CPET, Morbidity score, Major cancer surgery, Risk index score, Risk prediction tool, High-risk patient, Anaerobic threshold, Ventilatory equivalent, Surgery, Exercise, Preoperative evaluation

## Abstract

**Background:**

Morbidity and mortality risk prediction tools are increasingly being used as part of preoperative assessment of patients presenting for major abdominal surgery. Cardiopulmonary exercise testing (CPET) can predict which patients undergoing major abdominal surgery are at risk of complications. The primary objective of this study was to identify preoperative variables including those derived from CPET, which were associated with inpatient morbidity in high-risk patients following major abdominal cancer surgery. The secondary objective was to use these variables to derive and validate a morbidity risk prediction tool.

**Methods:**

We conducted a retrospective cohort analysis of consecutive adult patients who had CPET as part of their preoperative work-up for major abdominal cancer surgery. Morbidity was a composite outcome, defined by the Clavien-Dindo score and/or the postoperative morbidity survey (POMS) score which was assessed on postoperative day 7. A risk prediction tool was devised using variables from the first analysis which was then applied prospectively to a matched cohort of patients.

**Results:**

A total of 1398 patients were included in the first phase of the analysis between June 2010 and May 2017. Of these, 540 patients (38.6%) experienced postoperative morbidity. CPET variables deemed significant (*p* < 0.01) were anaerobic threshold (AT), maximal oxygen consumption at maximal exercise capacity (VO2 max), and ventilatory equivalent for carbon dioxide at anaerobic threshold (AT VE/VCO2). In addition to the CPET findings and the type of surgery the patient underwent, eight preoperative variables that were associated with postoperative morbidity were identified. These include age, WHO category, body mass index (BMI), prior transient ischaemic attack (TIA) or stroke, chronic renal impairment, diabetes mellitus, chronic obstructive pulmonary disease (COPD), and cancer stage. Both sets of variables were then combined to produce a validated morbidity risk prediction scoring tool called the Marsden Morbidity Index. In the second phase of the analysis, this tool was applied prospectively to 424 patients between June 2017 and December 2018. With an area under the curve (AUC) of 0.79, this new model had a sensitivity of 74.2%, specificity of 78.1%, a positive predictive value (PPV) of 79.7%, and a negative predictive value of (NPV) of 79%.

**Conclusion:**

Our study showed that of the CPET variables, AT, VO2 max, and AT VE/VCO2 were shown to be associated with postoperative surgical morbidity following major abdominal oncological surgery. When combined with a number of preoperative comorbidities commonly associated with increased risk of postoperative morbidity, we created a useful institutional scoring system for predicting which patients will experience adverse events. However, this system needs further validation in other centres performing oncological surgery.

## Introduction

As the population becomes more elderly, the incidence of cancer increases concomitantly with other comorbidities (Pedersen et al. 2016; Atella et al. 2019). Surgical resection forms the mainstay of treatment for most solid organ tumours (Bakos et al. 2018).

As a result, major cancer surgery is expected to account for a significant and disproportionate proportion of all healthcare spending in the developed world in the next 20 years (Sullivan et al. 2015). The ability to objectively predict morbidity preoperatively allows for better targeted resource allocation and optimisation; risk stratification and supports informed decision-making and consent (Moonesinghe et al. 2013).

While the in-hospital mortality following cancer surgery in modern day practice is low, patients still suffer from significant procedure-specific postoperative morbidity (Weiser and Gawande 2015; Morris et al. 2011; Endo et al. 2017). Adverse events following major abdominal cancer surgery are often linked to the severity of pre-existing comorbidities and the functional ability of patients to meet the extra metabolic demands required when undertaking complex surgery (Lee et al. 2013; Johnson et al. 2019). Cardiopulmonary exercise testing (CPET) is one method considered to be the gold standard, to objectively measure a patient’s cardiopulmonary function (Herdy et al. 2016). Poor performance on preoperative CPET has consistently been shown to be associated with morbidity following major abdominal cancer surgery (Older et al. 1999; Wilson et al. 2010; Lai et al. 2013; Junejo et al. 2014). In the UK, CPET is increasingly used for risk prediction as part of a comprehensive preoperative assessment, especially in “high-risk” patients prior to major surgery (Levett et al. 2018; Older et al. 2000). This study was primarily designed to investigate whether CPET, when combined with other commonly recorded preoperative variables, were associated with postoperative morbidity in a large mixed cohort of high-risk patients scheduled for major abdominal cancer surgery. The secondary aim was to devise a simple risk prediction model using these significant variables and then prospectively validate this model for our institution.

## Methods

### Primary objective

The primary objective was to identify which preoperative variables, including those derived from CPET, were independently associated with morbidity following major abdominal cancer surgery. This was studied as a retrospective cohort analysis of consecutive adult patients undergoing CPET as part of their preoperative work-up for major abdominal cancer surgery at a single high volume cancer centre, The Royal Marsden National Health Service Foundation Trust, London SW3 6JJ, between June 2010 and May 2017.

Inclusion criteria were all adult patients > 18 years of age who had a CPET assessment as part of their preoperative work-up for planned elective major abdominal cancer surgery. Ethical approval was obtained from the local institutional review board of the Royal Marsden NHS Foundation Trust and approved as a service evaluation (Reference SE443). The criteria for CPET in patients scheduled for major oncological abdominal surgery at the Royal Marsden NHS Foundation Trust are as follows:

- Patients > 18 years

And 1 or more of the following

- ≤ 4 metabolic equivalents*
- Significant cardiorespiratory comorbidities
- Do not meet any of the contraindications to CPET as stated by our institutional policy which is in line with the national guidance (Levett et al. 2018).

*It is based on guidance from the physical activity tables assessed by medical staff in pre-assessment (Ainsworth et al. 2011).

### Perioperative pathway

All “high-risk” patients studied had a preoperative assessment (assessment of fitness work-ups) prior to abdominal cancer surgery. They all had a CPET to assess their functional capacity, which was used to assess fitness for surgery and decision-making. Postoperatively, all patients were admitted to an intensive care or high dependency unit. Preoperative data was extracted from the electronic patient record (EPR) by the institution’s information support team who were not involved in the study. Preoperative comorbidities were documented in the patient’s EPR by a nurse and/or doctor at the pre-assessment and were collected based on the ICD-10 (International Statistical Classification of Diseases and Related Health Problems) (World Health Organisation 1994) definitions. CPET data was extracted from a contemporaneously held database by exercise physiologists who ran the tests.

### Secondary objective

The secondary objective was to create a simple risk score that assigns a point score to significant risk factors and then assign the different scores as a risk of morbidity using data from June 2010 to May 2017. This risk scoring system was then validated using prospectively collected data from an 18-month period between June 2017 and December 2018.

### Morbidity

Morbidity was evaluated as categorical data, using validated morbidity scoring system in the Clavien-Dindo (CD) complication grading system (Dindo et al. 2004) and the postoperative morbidity survey (POMS) (Grocott et al. 2007).

Morbidity was a composite outcome defined as follows:

- Clavien-Dindo score of ≥ 3
- Postoperative morbidity survey score of > 1 at postoperative day (POD) 7

A CD score ≥ grade 3 and/or POMS > 1 on POD 7 during the patient’s inpatient hospital stay were both classed as clinically significant complication (Clavien et al. 2009; Davies et al. 2013). Morbidity outcomes were assessed through analysis of the patient’s EPR and were recorded routinely by the Royal Marsden’s information team. If patients included in the study were discharged prior to POD 7, it was assumed that they had no morbidity.

### Cardiopulmonary exercise testing

Cardiopulmonary exercise testing was performed and reported by an accredited exercise physiologist. Testing was conducted using the standardised approach recommended by the American Thoracic Society (ATS) and American College of Physicians (ACCP) (American Thoracic Society; American College of Chest Physicians 2003a) in conjunction with the Perioperative Exercise Testing and Training Society (POETTS) guidelines (Levett et al. 2018). Exercise testing was conducted on an electromagnetically braked cycle ergometer (Ergoselect 200; Ultima CardiO2®; Medical Graphics Corp., St Paul, MN, USA) following resting spirometry. Ventilation and gas exchange were measured using a metabolic cart (Ultima™ CardiO2® gas exchange analysis system, MGC Diagnostics, Minnesota, USA). Routine physiological measurements of function included the following: work rate (Watts); spirometric parameters — minute ventilation (VE) and tidal volume (VT); metabolic gas exchange measurements — O2 consumption (VO2), CO2 production (VCO2), and respiratory exchange ratio (RER = VCO2/VO2); ventilatory equivalents for O2 (VE/VO2) and CO2 (VE/VCO2) at anaerobic threshold (AT); cardiovascular variables — heart rate, electrocardiogram (ECG), and NIBP; and respiratory variables — respiratory rate and oxygen saturation. The CPET data were analysed using CardioPerfect 1.6.2.1105 [Welch Allyn (UK) Ltd., Aston Abbotts, UK] and MedGraphics BreezeSuite 8.5.0.57SP3 (Medical Graphics Corp.). The AT was determined by the CPET physiologist and confirmed by a consultant anaesthetists experienced in CPET. Peak VO2 was also determined using the same 2-person technique and determined at maximal O2 consumption and at the point of peak exercise, determined by maximal Watts on the cycle ergometer.

### Analysis methods

The primary dataset was used to describe the patient characteristics in the morbidity and no-morbidity groups using counts and percentages for the categorical variables and mean/median and standard deviation or interquartile range. Binary logistic regression analysis method was then used in the univariate and multivariate settings to identify the morbidity risk factors. All variables were candidate in the multivariate model analysis. Backward stepwise method was used with a cut-off point (*p*-value < 0.01) for a variable to be included in the fitted multivariate model. Predicted probabilities were obtained for the primary and temporal validation datasets using the post estimation commands of the analysis software, which were then categorised at a cut-off point of 0.5 and summarized into binary classification table (observed and predicted morbidity) for a sensitivity analysis. Sensitivity, specificity, negative and positive predictive values, and classification accuracy of the model were calculated in the primary and validation datasets. Similarly, ROC (receiver operator characteristic) were fitted and AUC (area under the curve) values obtained. A nomograph was utilized to assign scores to the significant variables in the fitted multivariate logistic regression model to produce a graph that can be used clinically to identify the patient with high probability of morbidity risk. The preoperative status of the patients is applied against the nomograph variables to obtain the total scores, which can then be converted to probability to determine their morbidity risk level. The STATA version 13 was used for analysis (Kattan et al. 1998).

## Results

**Fig. 1:**
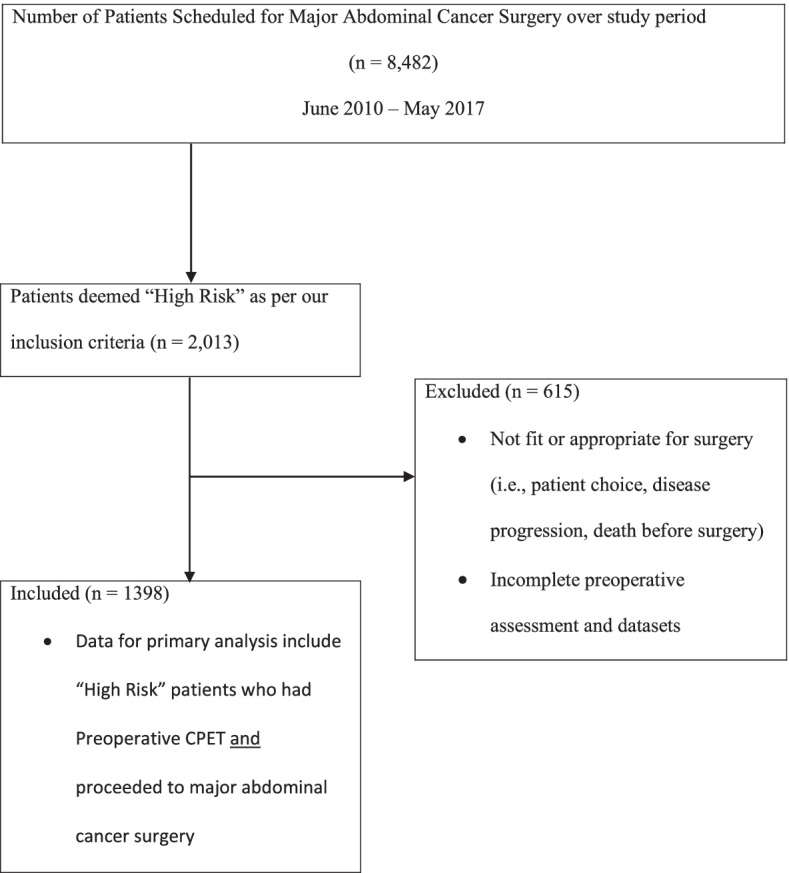
Modified CONSORT flow diagram for patients in the primary analysis. CPET, cardiopulmonary exercise testing

Figure 1 shows the flow of patients in the study. A total of 8482 patients were scheduled for major abdominal cancer surgery during the study period, of whom 2013 (23.7%) were deemed high risk. All procedures were either defined as major or major complex as defined by OPECS codes (Health and Social Care Information System 2015). Smaller abdominal procedures such as an appendectomy or cholecystectomy were not included unless they were part of a multivisceral resection. Of these, 615 patients were excluded from analysis either because CPET data were incomplete or they did not proceed to the intended major abdominal surgery following CPET. Reasons for not proceeding with surgery post CPET include patient declining surgery, death before the planned operation date, patient deemed unfit for surgery following a multidisciplinary team decision process, and, in the event, where the surgery was “open and close” due to unresectable disease. In total, 1398 patients (704 men and 694 women) underwent CPET followed by the intended abdominal surgery, and their data were included in the analysis.

Six-hundred and fifteen patients (*n* = 615) were excluded from the analysis. One-hundred and four (*n* = 104) patients were deemed too high risk for surgery following pre-assessment and CPET, and their CPET data is included in the Table 8 in Appendix. Fifty-three (*n* = 53) patients were excluded because of insufficient datasets. The remainder of patients (*n* = 458) was excluded for other reasons with the most common being decision for alternative oncological option (not related to fitness for surgery) and disease progression whereby surgery was not appropriate and surgery was not performed (for example patient choice; patient death).

Patient demographics and perioperative characteristics are summarised in Table 1. It also provides a summary of the CPET data measured for all 1398 patients included in the analysis. These were patients who had CPET and abdominal cancer surgery. The median age of these patients was 68 years (range: 21–89).

**Table 1:** Preoperative description of patients submitted to cardiopulmonary exercise testing (CPET) prior to major abdominal surgery and summary of CPET outcomes. *SD*, standard deviation; *IQR*, interquartile range

Variable	Overall patients (***n*** = 1398)
	**Median (range)**
Age (years)	
Median (range), (IQR)	68 (21–89), (65–78)
Body mass index (BMI)	
Mean (SD)	27.98 (5.87)
Median (range), (IQR)	28.0 (12.3–48.4), (24.3–32.0)
Anaerobic threshold (AT)	
Mean (SD)	10.79 (2.01)
Median (range), (IQR)	10.4 (5.5–19.9), (10.0–12.6)
VO2 max	
Mean (SD)	17.11 (5.74)
Median (range), (IQR)	15.8 (4.5–37.6), (13.9–18.9)
AT VE/VCO2	
Mean (SD)	35.68 (4.39)
Median (range), (IQR)	36 (20–48) [32–38],
Oxygen pulse	
Mean (SD)	9.63 (3.00)
Median (range), (IQR)	9 (2–14) [9–13],
	***n*(%)**
Age group	
≤ 50 years	164 (12)
51–70 years	722 (52)
> 70 years	512 (37)
Gender	
Female	694 (50)
Male	704 (50)
BMI standard categories	
< 18.5	203 (14)
18.5–24.99	199 (14)
≥ 25.0	996 (71)
Surgery type	
Robotic/laparoscopic	356 (25)
Open	1,042 (75)
American Society of Anaesthesiologists (ASA) score	
ASA 1	22 (2)
ASA 2	1,076 (77)
ASA 3	300 (21)
World Health Organisation (WHO) category	
0	735 (53)
1	565 (40)
2	53 (4)
3	43 (3)
4	2 (0.1)
Arrhythmia	
No	1,246 (89)
Yes	152 (11)
Documented cirrhosis	
No	1,307 (93)
Yes	91 (7)
Congestive cardiac failure	
No	1,358 (97)
Yes	40 (3)
Diabetic status	
Negative	1,034 (74)
Non-insulin	306 (22)
Insulin dependent	58 (4)
Chronic obstructive pulmonary disease (COPD)	
No	862 (62)
Yes	536 (38)
Previous cardiac event	
No	975 (70)
Yes	423 (30)
Prior transient ischaemic attack (TIA) or stroke	
No	1,269 (91)
Yes	129 (9)
Chronic renal impairment	
No	1,147 (82)
Yes	251 (18)
Number of procedures	
1	782 (56)
2	154 (11)
≥ 3	462 (33)
Cancer stage (TNM classification)	
Primary cancer	1,234 (88)
Local nodal metastases	140 (10)
Distant metastases	23 (2)
Surgical categories	
Hepatobiliary	228 (16)
Urology	340 (24)
General surgery	330 (24)
Colorectal/lower gastrointestinal (GI)	118 (8)
Upper gastrointestinal (GI)	234 (17)
Gynaecological	138 (10)
Sarcoma	9 (0.6)

### Primary and secondary analysis results

Five-hundred and forty patients (36.8%) experienced postoperative morbidity, a composite outcome defined as Clavien-Dindo score of ≥ 3 and/or the score for POMS in POMS-defined morbidity on POD 7. The CPET variables on univariate analysis associated with postoperative morbidity were AT, VO2 max, AT VE/VCO2, and oxygen pulse (Table 2).

**Table 2:** Univariate logistic regression analysis. *OR*, odds ratio. *95% CI*, 95% confidence interval

Variable	Morbidity ***n*** = 540	No morbidity ***n*** = 858	OR (95% ***CI***)	***p***-value
	**Median (range)**	**Median (range)**		
Age (years)				
Mean (SD)	68.36 (9.63)	66.79 (12.38)	1.01 (1.00–1.02)	0.013
Median (IQR)	68 (65–78)	68 (65–78)		
BMI				
Mean (SD)	27.79 (8.02)	28.10 (3.97)		
Median (IQR)	29 (18–35)	28 (26–32)	0.99 (0.97–1.01)	0.347
AT				
Mean (SD)	9.62 (2.00)	11.53 (1.64)		
Median (IQR)	9.6 (7.8–11.8)	11.1 (10.4–12.6)	0.54 (0.50–0.59)	< 0.001
VO2 max				
Mean (SD)	13.20 (4.35)	19.57 (5.11)		
Median (IQR)	12.9 (9.9–14.6)	18.9 (15.8–23.4)	0.71 (0.69–0.74)	< 0.001
AT VE/VCO2				
Mean (SD)	38.19 (3.70)	34.09 (4.03)		
Median (IQR)	36 (32–38)	35 (30–37)	1.32 (1.28–1.37)	< 0.001
Oxygen pulse				
Mean (SD)	9.46 (2.80)	9.75 (3.11)		
Median (IQR)	8.9 (6.7–13.2)	10.2 (6.6–13.2)	0.97 (0.93–1.00)	0.080
Possum score				
Mean (SD)	33.32 (6.10)	34.41 (9.97)		
Median (IQR)	34 (32–34)	34 (28–35)	0.99 (0.97–1.00)	0.023
	***n*(%)**	***n*(%)**		
Age group				
≤ 50 years	46 (28)	118 (72)	1	0.014
51–70 years	289 (40)	433 (60)	1.71 (1.18–2.48)	0.005
> 70 years	205 (40)	307 (60)	1.71 (1.17–2.51)	0.006
Gender				
Female	267 (38)	427 (62)	1	0.907
Male	273 (39)	431 (61)	1.01 (0.82–1.26)	
BMI standard categories				
< 18.5	171 (84)	32 (16)	29.00 (16.91–49.59)	< 0.001
18.5–24.99	31 (16)	168 (84)	1	< 0.001
≥ 25.0	338 (34)	658 (66)	2.78 (1.86–4.17)	< 0.001
Surgery type				
Robotic/laparoscopic	28 (8)	328 (92)	1	< 0.001
Open	512 (49)	530 (51)	11.32 (7.55–16.96)	
ASA score				
ASA 1	9 (41)	13 (59)	1	< 0.001
ASA 2	475 (44)	601 (56)	1.14 (0.48–2.69)	0.762
ASA 3 and 4	56 (19)	244 (81)	0.33 (0.14–0.81)	0.016
WHO category				
0	210 (29)	525 (71)	1	< 0.001
≥ 1	330 (50)	333 (50)	2.48 (1.99–3.09)	
Arrhythmia				
No	439 (35)	807 (65)	1	
Yes	101 (66)	51 (34)	3.64 (2.55–5.20)	< 0.001
Documented cirrhosis				
No	496 (38)	811 (62)	1	
Yes	44 (48)	47 (52)	1.53 (1.00–2.34)	0.050
Congestive cardiac failure				
No	506 (37)	852 (63)	1	< 0.001
Yes	34 (85)	6 (15)	9.54 (3.98–22.88)	
Diabetic status				
Negative	400 (39)	634 (61)	1	0.300
Non-insulin	123 (40)	183 (60)	1.07 (0.82–1.38)	0.634
Insulin dependent	17 (29)	41 (71)	0.66 (0.37–1.17)	0.155
COPD				
No	247 (29)	619 (71)	1	< 0.001
Yes	293 (55)	243 (45)	3.02 (2.40–3.76)	
Previous cardiac event				
No	302 (31)	673 (69)	1	< 0.001
Yes	238 (56)	185 (44)	2.87 (2.27–3.63)	
Prior TIA/stroke				
No	446 (35)	823 (65)	1	< 0.001
Yes	94 (73)	35 (27)	4.96 (3.31–7.43)	
Chronic renal impairment				
No	323 (28)	824 (72)	1	< 0.001
Yes	217 (86)	34 (14)	16.28 (11.09–23.90)	
Number of procedures				
1	252 (32)	530 (68)	1	< 0.001
≥ 2	288 (47)	328 (53)	1.85 (1.48–2.30)	
Cancer stage				
Primary	474 (38)	760 (62)	1	< 0.001
Local nodal metastases	45 (32)	95 (68)	0.76 (0.52–1.10)	0.148
Distant metastases	20 (87)	3 (13)	10.69 (3.16–36.17)	< 0.001
Surgical categories				
Hepatobiliary	85 (37)	143 (63)		
Urology	141 (41)	199 (59)		
General surgery	129 (39)	201 (61)		
Colorectal/lower GI	30 (25)	88 (75)		
Upper GI	99 (42)	135 (58)		
Gynaecological	52 (38)	86 (62)		
Sarcoma	3 (33)	6 (67)		

The nomogram demonstrates that CPET variables offer the most significant contribution in predicting and discriminating morbidity. However, the model is strengthened by the addition of the comorbidity variables which are significant and add an additional 0.173 to the significant CPET variables, for the AUC in discriminating morbidity.

Binary logistic regression analysis method was used in the univariate and multivariate settings to identify the morbidity risk factors. Backward stepwise method using (*p* < 0.01) was used to fit the multivariate model. Table 2 is the description of the morbidity and no-morbidity patients and summary of the univariate logistic regression results for all 1398 patients included in the primary analysis, while Table 3 is the summary of the output of the multivariate logistic regression results.

**Table 3:** Multivariate model output from backward stepwise selection model including only variables that are significant at (*p* < 0.01) in the multivariate model

Variable	OR (95% ***CI***)	***p***-value
VO2 max (continuous)	0.82 (0.77–0.86)	< 0.001
AT (continuous)	0.66 (0.61–0.71)	< 0.001
AT VE/VCO2 (continuous)	1.33 (1.26–1.40)	< 0.001
COPD		
No	1	0.001
Yes	1.99 (1.33–2.98)	
Chronic renal impairment		
No	1	< 0.001
Yes	7.50 (4.29–13.10)	
Age group		
> 70 years	1	0.001
51–70 years	0.35 (0.18–0.69)	0.003
< 50 years	0.25 (0.12–0.51)	< 0.001
Diabetic status		
Negative	1	0.002
Insulin	2.54 (1.52–4.23)	< 0.001
Non-insulin dependent	1.02 (0.98–1.06)	0.831
BMI categories		
< 18.5	6.98 (2.91–16.74)	< 0.001
18.5–24.99	1	< 0.001
≥ 25.0	1.72 (0.90–3.30)	0.102
Surgery type		
Robotic/laparoscopic	1	< 0.001
Open	10.80 (5.77–20.19)	
Cancer stage		
Primary	1	0.006
Local nodal metastases	1.61 (0.84–3.09)	0.153
Distant metastases	11.22 (2.31–54.66)	0.003
WHO category		
0	1	< 0.001
≥ 1	3.94 (2.58–6.03)	
Prior TIA/stroke		
No	1	0.002
Yes	3.07 (1.50–6.31)	

Of the 540 patients who suffered from morbidity, 413 (76.8%) scored POMS ≥ 1 on POD 7, 46 (8.5%) experienced a ≥ 3 CD complication, and 81 (15%) had both a POMS ≥ 1 and experienced a ≥ 3 CD complication (Table 4).

**Table 4:** Fitted model binary classification table (observed and predicted morbidity)

Probabilities	Morbidity *n* (%)	No morbidity *n* (%)	Total
≥ 0.5 (morbidity)	462 (86)	60 (7)	522
< 0.5 (no morbidity)	78 (14)	798 (93)	876
Total	540	858	1,398

### Fitted model classification ability

Table 5 shows the difference in the prediction strength of the model with and without the CPET variables, giving the model a prediction strength (pseudo R^2^) of 0.59. This shows that when the significant CPET variables on multivariable analysis are removed from the model, its predictive and discriminatory ability for serious morbidity is markedly reduced. This supports the value of CPET variables as part of a risk predictor for morbidity for our patient cohort.

**Table 5:** Comparison in prediction strength of the model with and without CPET variables

	With CPET variables	Without CPET variables
AUC	0.81	0.64
Sensitivity	75.7%	65.4%
Specificity	73.0%	61.5%
Positive predictive value (PV)	78.5%	60.3%
Negative PV	71.2%	59.8%
Correctly classified	80.2%	62.5%

### Temporal validation: using the validation dataset

#### Description of patients in the validation dataset

Four-hundred and twenty-four patients who had surgery in the year June 2017–December 2018 were used for the model (temporal) validation. The median age of these patients was 68 years (range: 49–79 and *IQR*: 68–69) (Table 6).

**Table 6:** Temporal dataset binary classification table (observed and predicted morbidity)

Probabilities	Morbidity *n* (%)	No morbidity *n* (%)	Total
≥ 0.5 (morbidity)	192 (84)	43 (22)	235
< 0.5 (no morbidity)	36 (16)	153 (78)	189
Total	228	196	424

#### Model classification ability

AUC = 0.79

Sensitivity = 74.2

Specificity = 78.1

Positive PV = 79.7

Negative PV = 79.0

Correctly classified = 71.4

**Fig. 2:**
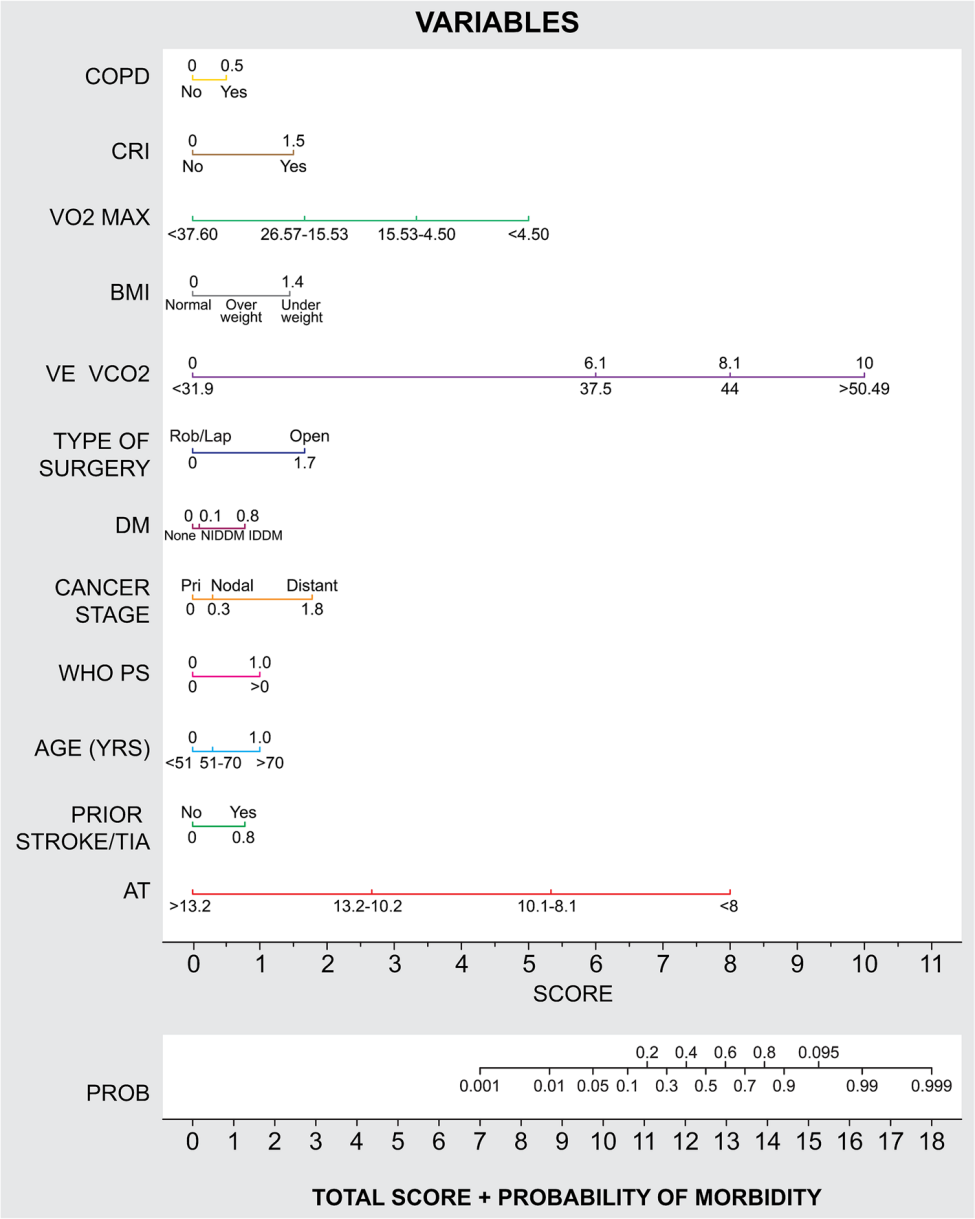
Fitted model variables — nomograph. These are COPD, chronic obstructive pulmonary disease. CRI, chronic renal impairment. VO2 max, maximal oxygen consumption at maximal exercise capacity. BMI, body mass index. VE/VCO2 — ventilatory equivalent for carbon dioxide; VE/VCO2 values in this diagram refers to VE/VCO2 taken at anaerobic threshold (AT VE/VCO2). Rob/Lap, robotic-assisted or laparoscopic-assisted surgery. DM, diabetes mellitus. NIDDM, non-insulin-dependent DM. IDDM, insulin-dependent DM. Pri, primary. WHO PS, World Health Organisation point score. TIA, transient ischaemic attack. CPET, cardiopulmonary exercise testing. AT, anaerobic threshold

Table 7 shows the variables from Table 3 which are used to construct a nomogram to predict morbidity. The nomogram is displayed in Fig. 2. Each of the variables is assigned a predicted score based on its contribution towards morbidity. The total predicted score on the nomogram in Fig. 2 corresponds to a probability of morbidity at the foot of the nomogram.

**Table 7:** Nomograph variables predicted scores

Nomograph variables	Predicted scores	Nomograph variables	Predicted scores
**COPD**		**Chronic renal impairment**	
No	0.0	No	0.0
Yes	0.5	Yes	1.5
**CPET: VO2 max**		**BMI categories**	
37.60	0.0	Normal	0.0
26.57	1.6	Underweight	1.4
15.53	3.3	Overweight	0.4
4.50	4.9		
		**Age groups**	
**CPET: AT VE/VCO2**		< 51 years	1.0
38.67	4.2	51–70 years	0.3
41.67	6.1	> 70 years	0.0
43.87	8.1		
> 45.00	10.0	**Type of surgery**	
		Robotic laparoscopy	0.0
**WHO point score**		Open	1.7
WHO = 0	0.0		
WHO > 0	1.0	**Diabetes**	
		Negative	0.0
**Prior TIA stroke**		Non-insulin	0.1
No	0.0	Insulin dependent	0.8
Yes	0.8		
		**CPET: AT**	
**Cancer stage**		> 1 3.2	0
Primary	0.0	10.2	2.6
Nodal metastases	0.3	8.1	5.2
Distant metastases	1.8	< 8.0	8.0

## Discussion

In this retrospective cohort of high-risk patients presenting for major abdominal cancer surgery, we found that postoperative morbidity was significantly associated with twelve variables: age, BMI, WHO status, cancer stage (TNM classified), CPET-generated data (AT, VO2 max, and AT VE/VCO2), pre-existing comorbidities (chronic renal impairment, COPD, diabetes mellitus, and a previous history of TIA or stroke), and whether a minimally invasive or an open approach was undertaken. These variables were shown to have good strength in discriminating postoperative morbidity in a prospective group of major abdominal cancer surgical patients. Using a scoring system based on the significance of each of these variables on postoperative morbidity, a simple risk scoring system called the “Marsden Morbidity Index” was devised. This index can be used in our institution to predict morbidity in patients scheduled for major abdominal surgery as a means of aiding decision-making, consent, and resource allocation. These variables were broadly reflective of the functional measures which deemed the need for CPET prior to surgery in this patient cohort.

The CPET variables we found to be associated with morbidity were in keeping with findings from previous studies where CPET was evaluated as a risk prediction tool in major abdominal surgery (Snowden et al. 2010; American Thoracic Society; American College of Chest Physicians 2003b; Hennis et al. 2011). Our study demonstrated that AT and VO2 max were significant (*p* < 0.001) variables at the multivariate analysis level and predictive of poor surgical outcomes. In the perioperative context, both have been shown to be strong predictors of postoperative complications and mortality in a number of cohorts analysing outcomes post major abdominal and thoracic surgery (Smith et al. 2009; Nagamatsu et al. 2015; Brunelli et al. 2014). West et al. (West et al. 2014) conducted a prospective blinded observational study to investigate for any association between CPET findings and postoperative morbidity after major colonic surgery. Patients who suffered postoperative complications had significantly lower oxygen uptake at lactate threshold, lower VO2 at peak, and higher AT VE/VCO2. These variables were found to be independently predictive of morbidity post rectal cancer surgery and major colonic surgery. Lee et al. (Lee et al. 2013) demonstrated a significant association between preoperative oxygen consumption on a 6-min walk test and postoperative medical complications (*p* < 0.01) post elective colorectal resection.

In our analysis, VE/VCO2 at anaerobic threshold had the strongest weighting in the model for postoperative morbidity in major abdominal surgery. This is a measure of ventilatory efficiency and is elevated in conditions such as heart failure, pulmonary embolism, and chronic lung disease (Wilson et al. 2019). It is thus unsurprising that this variable is so strongly associated with morbidity. According to Junejo et al. (Junejo et al. 2012), CPET findings for preoperative risk assessment before pancreatoduodenectomy showed VE/VCO2 at AT to be the only CPET variable independently associated with postoperative morbidity, with an AUC of 0.65 (95% *CI* 0.53–0.77). Similar to our study, CPET was applied in patients deemed high risk, and POMS scores were used to assess postoperative morbidity. An AT VE/VCO2 of ≥ 34.5 ml/kg/min was found to have a specificity of 84% and a sensitivity of 47%, with a PPV of 76% and an NPV of 60%, for POMS-defined morbidity.

Anaerobic threshold (VO_2_ at AT) was a significant CPET variable associated with postoperative morbidity in this analysis of high-risk patients undergoing major abdominal cancer surgery. This is consistent with one of our previous studies that demonstrated VO2 at *AT* < 10.2 ml/kg/min as a significant predictor of POMS-defined morbidity on POD 3 in patients undergoing major hepatic resection (Kasivisvanathan et al. 2015). Peak VO2 was also shown to be significantly associated with morbidity, consistent with other multiple studies (Older and Levett 2017; Andrade and Lopes 2015). It should be noted that VO_2_ at AT and VO2 peak usually have significant interactions so this finding is not entirely unexpected.

Objective risk identification and stratification are pivotal in linking preoperative comorbidities to risk-adapted intraoperative approaches and targeted postoperative care pathways. There are multiple grading and risk stratification tools currently in use for surgical patients. However, many of these systems are largely subjective and do not take into account any objective functional status or surgery-related factors.

The “Marsden Morbidity Index” was developed on the strong advocacy for CPET as an objective risk prediction tool based on current evidence and literature (Stringer 2010). Our aim was to combine CPET variables with premorbid variables to increase acuity in risk prediction. The use of CPET was supported by our study which demonstrated that the incorporation of CPET variables into a risk prediction tool that also takes other significant clinical variables into account creates a stronger risk prediction model. The comorbidities we identified are strongly validated in other risk scoring systems (Van Diepen et al. 2014; Barnett and Moonesinghe 2011; Stones and Yates 2019; Wong et al. 2017) currently in use, reflecting the precision of this new model. For this model, the AUC to discriminate morbidity was 0.81 and 0.79 in the fitted model binary classification and the temporal validation model respectively.

Preoperative variables deemed significant in the generation of the “Marsden Morbidity Index” risk prediction tool can be further sub-grouped into baseline parameters (age, BMI, WHO category, and TNM-classified cancer stage) and chronic conditions (COPD, diabetes mellitus, chronic renal impairment, and a previous history of TIA or stroke). The majority of these variables have been strongly validated in multiple risk prediction scores, like CHA_2_DS_2_-VASc (Van Diepen et al. 2014), p-POSSUM (Barnett and Moonesinghe 2011), Lee’s Revised Cardiac Risk Index (Stones and Yates 2019), and SORT (Wong et al. 2017) where one or more of these pre-existing variables are incorporated in a multifactorial risk-score calculation tool.

An interesting finding of our analysis showed that a low BMI scored higher than a high BMI. The effect of BMI on postoperative complications have been long studied with weight taken as a reflection of general health status from a broader perspective. From a preoperative evaluation, it reflects preoperative nutritional status, functional status, and the presence of comorbidities. While obesity is generally assumed to be a risk factor for postoperative adverse events, there is no convincing data to support this assumption (Tjeertes et al. 2015). A study published by Tjeertes et al. (Tjeertes et al. 2015) to seek more understanding of the obesity paradox revealed that while obesity alone is a significant risk factor for wound infection, more surgical blood loss, and a longer operation time, being obese is also associated with improved long-term survival. Complication and mortality rates were found to be significantly worse for underweight patients, who were most at risk of major postoperative complications, including long-term mortality. We also know from current literature that many of the CPET variables, like peak or VO_2_ max, are highly correlated with muscle mass (Sugie et al. 2017; Kim et al. 2016). While there is no available data on the direct comparison between CPET outcomes for high versus low BMI in cancer patients, the findings are a cause of concern that patients with low BMI are likely to perform equivocally if not worse than obese patients.

In addition, our study featured patients who underwent an open laparotomy were more likely to suffer from postoperative complications (*p* < 0.001) when compared to minimally invasive surgery, i.e. robotic assisted or laparoscopy. These findings are in keeping with the literature where the unique benefits and superiority of minimally invasive procedures over open procedures in selected patients have been shown (Buia et al. 2015). A systematic review and meta-analysis by Wang et al. comparing the two approaches for pancreatico-duodenectomy showed significant reductions in estimated blood loss, postoperative haemorrhage, transfusion rate, wound infection, and length of hospital stay (Wang et al. 2017). Similar findings from comparison between laparoscopy and laparotomy for rectal cancer include reductions in postoperative pain, length of stay, incisional hernia, adhesive bowel obstruction, wound complications, and mortality (Kavalukas et al. 2020). The use of robotic-assisted surgery in the management of cancer continues to increase with numerous evidence in the literature of a shorter convalescence period postoperatively (Ashrafian et al. 2017).

In conclusion, we found the CPET variables of AT, VO2 max, and AT VE/VCO2, and a number of preoperative baseline demographics and comorbidities, commonly associated with increased risk of postoperative morbidity, were shown to be associated with postoperative surgical morbidity following major abdominal oncological surgery. Our study shows that the incorporation of CPET variables into a risk prediction tool produces a model with a strong ability to discriminate postoperative complications when morbidity was assessed using a combination of the Clavien–Dindo classification scoring system and the postoperative morbidity survey.

While this model has helped us create a useful institutional tool for perioperative risks, it needs further validation in other centres performing oncological surgery. In addition, further work is required to prospectively compare the Marsden Morbidity Index’s ability to predict morbidity with other validated risk calculators, and the retrospective nature of this study and real-time evolution of current calculators prevented this for the purposes of this study. To our knowledge, the Marsden Morbidity Index is unique in that it is one of only a few validated risk-scoring tools that directly incorporate CPET variables as part of their algorithms to predict perioperative outcomes.

### Strengths and limitations

The strength of this study is the large number of “high-risk” cancer patients that were studied (*n* = 1398). This makes it one of the largest published datasets looking at the association of CPET on postoperative surgical morbidity. This was a strongly validated study, and the result reflects the high-risk cohort of patients that present to the Royal Marsden Hospital as a tertiary oncological centre.

One of the major limitations of our study is that only high-risk patients based on our institutional criteria had CPET. The ideal study design would be that all patients had CPET to limit bias in the population studied. The risk calculator is thus only valid on our high-risk patient cohort. Nonetheless, when looking at real-world use of CPET, most published data is from a high-risk cohort of patients extracted from a general surgical population. The authors chose a POMS score of > 1 on POD 7, based on similar studies using POMS scores on this day as their preferred measure to discriminate morbidity in similar major surgical cohorts (Wong et al. 2017). Another major limitation was that the study did not account for individual surgical specialities, patient pathways, and the fact that the study occurred over a 10-year period where perioperative practices changed. Despite this, the variables initially derived as being associated with morbidity were strongly validated in predicting and discriminating (*AUC* 0.79) in the prospectively studied population. This suggests that despite a number of important factors not being accounted for in the preoperative variables, the model is a strong tool for our population. We would be interested in implementing its use in our institution which may provide further validation of the data.

## Data Availability

The datasets generated during and/or analysed during the current study are available in the Royal Marsden Hospital, and the datasets during and/or analysed during the current study are available from the corresponding author on reasonable request.

## Appendix

Table 8.

**Table 8:** CPET data for patients excluded from analysis because they were deemed too high risk following preoperative assessment and CPET

Variable	Overall patients (***n*** = 104)
	Median (range)
Age (years)	
Median (range), (IQR)	78 (55–99)
Body mass index (BMI)	
Mean (SD)	19.98 (7.87)
Median (range), (IQR)	18.0 (11.3–51.3), (20.3–25.4)
Anaerobic threshold (AT)	
Mean (SD)	6.79 (3.01)
Median (range), (IQR)	7.4 (3.5–10.8), (7.4–8.7)
VO2 max	
Mean (SD)	13.11 (2.24)
Median (range), (IQR)	12.8 (4.3–21.6), (12.4–15.6)
AT VE/VCO2	
Mean (SD)	38.28 (2.39)
Median (range), (IQR)	37 (30–58) [32–43],
Oxygen pulse	
Mean (SD)	6.63 (5.40)
Median (range), (IQR)	7.12 (3.41–12), (5.7–11)
